# Supplementary material for: Metassembler: merging and optimizing de novo genome assemblies
Source: Genome Biol. 2015 Sep 24;16:207. doi: 10.1186/s13059-015-0764-4 (PMC4581417; doi:10.1186/s13059-015-0764-4)
Supplement: Additional file 1: — SupplementaryFile_v4.docx: Supplementary Notes, Figures, and Tables. (DOCX 795 kb) [file 13059_2015_764_MOESM1_ESM.docx]

**Metassembler: Merging and optimizing de novo genome assemblies**

Alejandro Hernandez Wences, Michael C. Schatz

Table of Contents

Supplemental Note 1. Metassembler Parameters Used 3

Supplemental Note 1a. Assemblathon1 Parameters 3

Supplemental Note 1b. Assemblathon2 Parameters 4

Supplemental Note 2. Description of Supplemental files 6

Supplemental Note 3. PCA analysis of Assemblathon 1 metrics 7

Supplemental Note 4. ICA analysis of Assemblathon 1 metrics 8

Supplemental Table 1. Assemblathon 1 input assemblies information. 9

Supplemental Table 2. Principal Components of the Assemblathon 1 metrics 10

Supplemental Table 3. Independent components of the Assemblathon 1 metrics 11

Supplemental Table 4. Assemblathon 2 input assemblies information. 12

Supplemental Figure 1. Assemblathon 1 metassembly statistics 13

Supplemental Figure 1a. Metassembly scaffold NG50 size distribution. 13

Supplemental Figure 1b. Metassembly GAGE-corrected scaffold NG50 size distribution. 14

Supplemental Figure 1c. Metassembly contig NG50 size distribution. 15

Supplemental Figure 1d. Metassembly GAGE-corrected contig NG50 size distribution. 16

Supplemental Figure 1e. Metassembly Duplicated Reference Bases distribution. 17

Supplemental Figure 1f. Metassembly Compressed Reference Bases distribution. 18

Supplemental Figure 1g. Metassembly Translocations distribution. 19

Supplemental Figure 1h. Metassembly Relocations distribution. 20

Supplemental Figure 1i. Metassembly Inversions distribution. 21

Supplemental Figure 1j. Metassembly Overall Z-score distribution. 22

Supplemental Figure 2. Change in CE-statistic at indel breakpoint positions for the Assemblathon 1 metassemblies 23

Supplemental Figure 3. Assemblathon 1 Comparison between Metassembler and GAM 24

Supplemental Figure 3a. Comparison of Metassembly and GAM-NGS scaffold NG50 size distribution. 24

Supplemental Figure 3b. Comparison of Metassembly and GAM-NGS GAGE-corrected scaffold NG50 Z-score distribution. 25

Supplemental Figure 3c. Comparison of Metassembly and GAM-NGS contig NG50 Z-scores distribution. 26

Supplemental Figure 3d. Comparison of Metassembly and GAM-NGS GAGE-corrected contig NG50 Z-scores distribution. 27

Supplemental Figure 3e. Comparison of Metassembly and GAM-NGS Duplicated Reference Bases distribution. 28

Supplemental Figure 3f. Comparison of Metassembly and GAM-NGS Compressed Reference Bases distribution. 29

Supplemental Figure 3g. Comparison of Metassembly and GAM-NGS Translocations distribution. 30

Supplemental Figure 3h. Comparison of Metassembly and GAM-NGS Relocations distribution. 31

Supplemental Figure 3i. Comparison of Metassembly and GAM-NGS Inversions distribution. 32

Supplemental Figure 3j. Comparison of Metassembly and GAM-NGS Overall Z-score distribution. 33

Supplemental Figure 4. Boxplots of Overall Z-scores computed using only ICA-selected metrics, for all Assemblathon 1 metassamblies. 34

Supplemental Figure 4a. Overall Z-scores using only the top 50% ICAs 34

Supplemental Figure 4b. Overall Z-scores using only the top 80% ICAs 35

Supplemental Figure 5. Assemblathon 2: Change in CE-statistic at indel breakpoint positions 36

Supplemental Figure 5a. Fish: Change in CE-statistic at indel breakpoint positions 36

Supplemental Figure 5b. Bird: Change in CE-statistic at indel breakpoint positions 37

Supplemental Figure 5c. Snake: Change in CE-statistic at indel breakpoint positions 38

## Supplemental Note 1. Metassembler Parameters Used

### Supplemental Note 1a. Assemblathon1 Parameters

For all Assemblathon 1 assemblies and metassemblies we used the 3Kb mate-pair library available on the Assemblathon web page for the computation of the CE-statistic.

Mapping: *bowtie2 --minins 2000 --maxins 4000 --threads 16*

CE statistic: *mateAn -A 2500 -B 3500.*

WGA: *nucmer –maxmatch -l 50 -c 300*.

Merges: *asseMerge* with default options

Runtime Requirements:

Bowtie alignment: ~15 min

CEstat computation: ~21 min

Nucmer WGA: ~5 min

asseMerge: ~92 s

meta2fasta: ~5 s

Peak RAM Requirement: 2.6GB

### Supplemental Note 1b. Assemblathon2 Parameters

**I. Fish:**

For all Fish assemblies and metassemblies we used the available 2Kb mate-pair libraries:

801KYABXX.2 and 801KYABXX.3

Mapping: *bowtie2 --maxins 3000 --minins 1000 --threads 16*

CE-statistic: *mateAn -A 1500 -B 2600*

WGA: *nucmer –maxmatch -l 50 -c 300*

Merges: *asseMerge* with default options

Runtime Requirements:

Bowtie alignment: ~6.2 h

CEstat computation: ~2.6 h

Nucmer WGA: ~57 h

asseMerge: ~45 min

meta2fasta: ~70 s

Peak RAM requirement: 36GB

**II. Bird:**

For the Bird species we used the 2Kb libraries:

110503_I266_FCB05AKABXX_L5_PARprgDAPDWBAPE and

110515_I260_FCB0618ABXX_L5_PARprgDAPDWBAPE

Mapping: *bowtie2 --maxins 3000 --minins 1000 --threads 16*

CE-statistic: *mateAn -A 1500 -B 2600*

WGA: *nucmer –maxmatch -l 50 -c 300*

Merges: *asseMerge with default options*

Runtime Requirements:

Bowtie alignment: ~3 h

CEstat computation: ~3 h

Nucmer WGA: ~4 h

asseMerge: ~10.5 min

meta2fasta: ~45 s

Peak RAM requirement: 37GB

**Snake:**

For the Snake species we used the 2Kb libraries:

110405_EAS192_0222_FC70N35AAXX_lane1.fastq.gz

110405_EAS192_0222_FC70N35AAXX_lane2.fastq.gz

110405_EAS192_0222_FC70N35AAXX_lane3.fastq.gz

Mapping: *bowtie2 --maxins 3000 --minins 1000 --threads 16*

CE-statistic: *mateAn -A 1500 -B 2600*

WGA: *nucmer –maxmatch -l 50 -c 300*

Merges: *asseMerge with default options*

Runtime Requirements:

Bowtie alignment: ~1.6 h

CEstat computation: ~2.3 h

Nucmer WGA: ~5.3 h

asseMerge: ~30 min

meta2fasta: ~63 s

Peak RAM requirement: 46GB

##

## Supplemental Note 2. Description of Supplemental files

1. A1MetEventsTable.txt: Reported metassembly events (i.e. modifications to the primary assembly such as gaps closed, number of scaffold links, etc) for all Assemblathon1 metassemblies at each merging step
2. A1metricTable.txt: GAGE reported assembly evaluation metrics for all Assemblathon1 metassemblies at each step.
3. A1Zscores.txt: Zscores for all metrics and Overall Zscore for all metassemblies and input assemblies
4. A2MetEventsTable.txt: Reported metassembly events for all Assemblathon2 metassemblies of the three species at each merging step.
5. A2metricTable.txt: REAPR, CEGMA and contiguity statistics reported for all Assemblathon2 metassemblies of the three species at each step.
6. A2Zscores.txt: Zscores for all metrics and Overall Zscore for all metassemblies and input assemblies of the three species.

## Supplemental Note 3. PCA analysis of Assemblathon 1 metrics

PCA is a dimensionality reduction technique that finds the directions in the variable space, assembly evaluation metrics in our case, that have zero correlation and account for the most variance. ICA is a technique that looks for a linear representation of nongaussian data where the components are constrained to be independent, or as independent as possible. Together these analyses can determine if the metrics are redundant, and if so, eliminate the influence of redundant metrics on the composite rank.

A PCA analysis on a dataset consisting of *n* variables retrieves *n* components; however we are interested in selecting only those components that explain the majority of the observed variance (at least 90%), thus reducing the number of variables needed to describe the data without significant loss of information. The first component explains the largest amount of variance; the second component explains the second largest proportion of variance with the restriction of being orthogonal to the first component, and so on. With this approach, the set of ten input variables was reduced to just three principal components that explain more than 90% of the variance (Sup Table 2). This indicates that the measured metrics are highly correlated, and that the data should undergo ICA analysis to remove dependencies.

## Supplemental Note 4. ICA analysis of Assemblathon 1 metrics

The goal of ICA is to find the source independent non-guassian variables *si* that have been linearly combined to produce observed data **x**, that is **x** = **As**. In particular, ICA looks for an un-mixing matrix **W** (i.e. the inverse of **A**) such that **s** = **Wx** with si = **wi x** having the least gaussian distribution possible; thus converting ICA into a numerical optimization problem. One of the typical measures of gaussianity to maximize is the fourth-order cumulant or kurtosis. Gaussian random variables have a kurtosis value of zero whereas most non-gaussian random variables have a non-zero kurtosis.

In our analysis we selected only those components with a kurtosis value in the top 50% and 80% of the kurtosis distribution and from each of these components we retrieved the metric that contributed the most to the linear combination as previously used (Vezzi *et al.*, 2012). The resulting subset of statistically most informative metrics were: 1) Inversions, 2) Compressed Reference Bases, 3) Missing Reference Bases, and 4) Relocations for the top 50% components, plus 5) Duplicated Reference Bases for the top 80%. Using just these subsets of quality metrics, the same dependency between initial assembly and final composite score was observed (Supplementary figure 2).

Supplemental Reference:

Vezzi F, Narzisi G, Mishra B (2012) Feature-by-Feature – Evaluating De Novo Sequence Assembly. PLoS ONE 7(2): e31002. doi:10.1371/journal.pone.0031002

## Supplemental Table 1. Assemblathon 1 input assemblies information.

| Assemblathon 1 ID | Software | Assemblathon 1 Z-score rank | Contig N50  rank / size | Scaffold N50  rank / size |
| --- | --- | --- | --- | --- |
| Broad | ALLPATHS-LG | 1 | 2 / 208 Kb | 2 / 8.28 Mb |
| BGI | SOAPdenovo | 2 | 1 / 342 Kb | 5 / 1.7 Mb |
| WTSI-S | SGA | 3 | 4 / 26.3 Kb | 3 / 2.7 Mb |
| DOEJGI | Meraculous | 4 | 5 / 15 Kb | 1 / 9.07 Mb |
| CSHL | Celera, Bambus2 | 5 | 3 / 75.5 Kb | 4 / 2.46 Mb |

##

## Supplemental Table 2. Principal Components of the Assemblathon 1 metrics

|  | PCA1 | PCA2 | PCA3 |
| --- | --- | --- | --- |
| Scaffold NG50 | -0.267 | -0.356 | -0.292 |
| Contig NG50 | -0.416 |  | -0.220 |
| GC-Scaffold NG50 | -0.113 | -0.354 | 0.769 |
| GC-Contig NG50 | -0.395 | 0.204 | 0.216 |
| Duplicated Reference Bases | -0.428 |  |  |
| Compressed Reference Bases | 0.411 | -0.177 |  |
| Translocations | 0.286 | 0.367 | 0.283 |
| Relocations |  | 0.478 | -0.129 |
| Inversions |  | 0.51 |  |
| Missing Reference Bases | 0.381 | -0.216 | -0.334 |
| Cumulative Variance  (% of Total Variance) | 50.2% | 86% | 93% |

First three principal components along with their cumulative variance. Only significant weights for each component are shown.

## Supplemental Table 3. Independent components of the Assemblathon 1 metrics

|  | ICA 1 | ICA 2 | ICA 3 | ICA 4 | ICA 5 | ICA 6 | ICA 7 | ICA 8 | ICA 9 | ICA 10 |
| --- | --- | --- | --- | --- | --- | --- | --- | --- | --- | --- |
| Scaffold NG50 | 0.13 | 0.35 | -0.42 | 0.31 | -0.44 | 0.31 |  | 0.52 | -0.14 |  |
| Contig NG50 | 0.19 |  | -0.19 |  | -0.14 | 0.12 | 0.19 | -0.3 | 0.68 | -0.55 |
| GC-Scaffold NG50 | -0.17 | -0.12 | -0.38 | -0.11 | -0.26 |  | 0.14 | -0.12 | 0.41 | 0.72 |
| GC-Contig NG50 | -0.35 | 0.19 | -0.24 | -0.38 | -0.37 | -0.3 | -0.49 | -0.28 | -0.19 | -0.24 |
| Duplicated Reference Bases | 0.495 | 0.446 |  |  | -0.13 |  | 0.19 | -0.57 | -0.33 | 0.24 |
| Compressed Reference Bases | 0.1 | 0.62 | 0.14 | -0.27 | 0.39 |  | -0.36 | 0.23 | 0.38 | 0.2 |
| Translocations | -0.38 | 0.4 | -0.11 | -0.2 | 0.12 | -0.24 | 0.72 | 0.17 | -0.13 | -0.13 |
| Relocations |  | -0.14 |  | -0.64 |  | 0.74 |  |  | -0.11 |  |
| Inversions | -0.606 | 0.222 |  | 0.468 | 0.186 | 0.431 | -0.1 | -0.356 |  |  |
| Missing Reference Bases | -0.17 | 0.1 | 0.74 |  | -0.6 |  |  | 0.11 | 0.18 |  |
| Kurtosis | 11.57 | 9 | 6.7 | 5.96 | 5.62 | 4.98 | 3.79 | 1.76 | 1.57 | 1.57 |

Independent components along with their kurtosis value. Only significant weights for each component are shown.

## Supplemental Table 4. Assemblathon 2 input assemblies information.


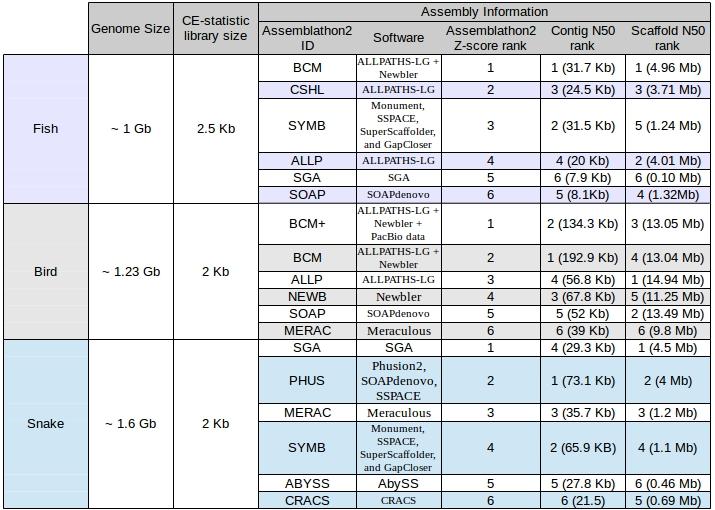


##

## Supplemental Figure 1. Assemblathon 1 metassembly statistics


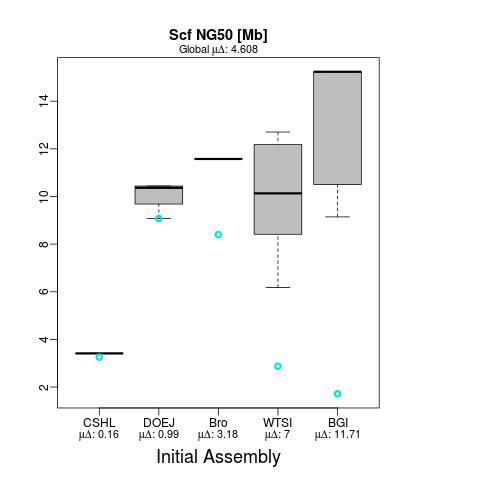


### Supplemental Figure 1a. Metassembly scaffold NG50 size distribution.

Distributions of scaffold NG50 sizes (in mega bases) reported for the set of all the 120 metassemblies, grouped by initial assembly. Blue circles indicate the corresponding value of the initial assembly. Below each initial assembly, the mean difference between the final metas1. Asssembly and the initial assembly (μ𝚫) is shown in mega bases. The global mean difference is also shown at the top.


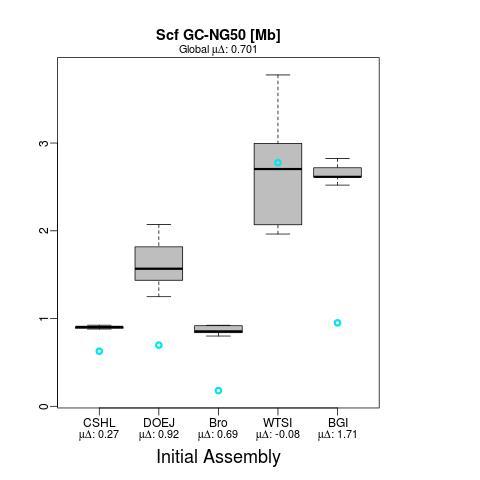


### Supplemental Figure 1b. Metassembly GAGE-corrected scaffold NG50 size distribution.

Distributions of GAGE-corrected scaffold NG50 sizes (in mega bases) reported for the set of all the 120 metassemblies, grouped by initial assembly. Blue circles indicate the corresponding value of the initial assembly. Below each initial assembly, the mean difference between the final metassembly and the initial assembly (μ𝚫) is shown in mega bases. The global mean difference is also shown at the top.


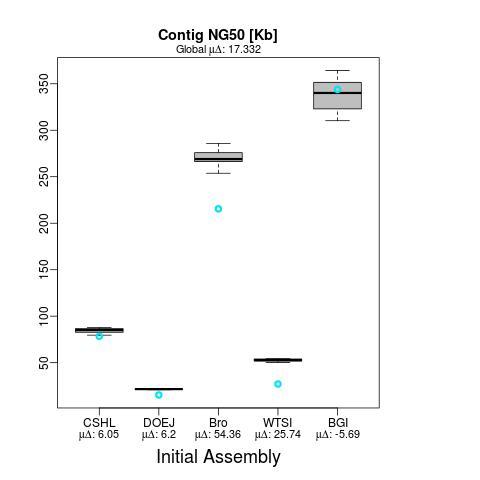


### Supplemental Figure 1c. Metassembly contig NG50 size distribution.

Distributions of contig NG50 sizes (in kilobases) reported for the set of all the 120 metassemblies, grouped by initial assembly. Blue circles indicate the corresponding value of the initial assembly. Below each initial assembly, the mean difference between the final metassembly and the initial assembly (μ𝚫) is shown in kilo bases. The global mean difference is also shown at the top.


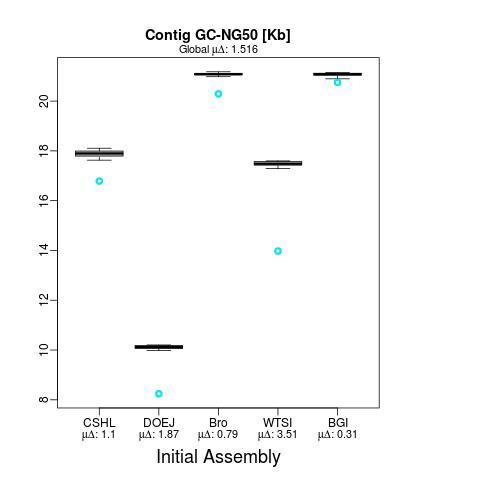


### Supplemental Figure 1d. Metassembly GAGE-corrected contig NG50 size distribution.

Distributions of GAGE-corrected contig NG50 sizes (in kilo bases) reported for the set of all the 120 metassemblies, grouped by initial assembly. Blue circles indicate the corresponding value of the initial assembly. Below each initial assembly, the mean difference between the final metassembly and the initial assembly (μ𝚫) is shown in kilo bases. The global mean difference is also shown at the top.


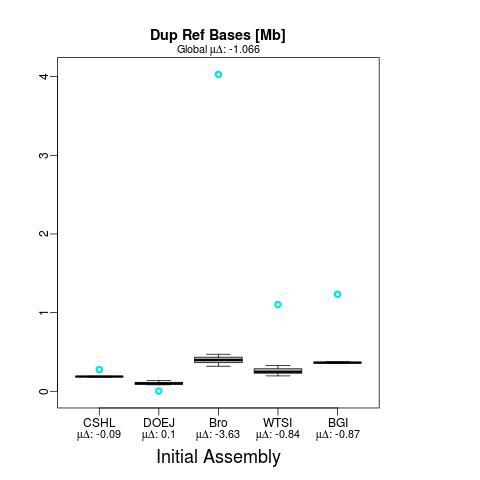


### Supplemental Figure 1e. Metassembly Duplicated Reference Bases distribution.

Distributions of the number of Duplicated Reference Bases (in mega bases) reported for the set of all the 120 metassemblies, grouped by initial assembly. Blue circles indicate the corresponding value of the initial assembly. Below each initial assembly, the mean difference between the final metassembly and the initial assembly (μ𝚫) is shown in mega bases. The global mean difference is also shown at the top.


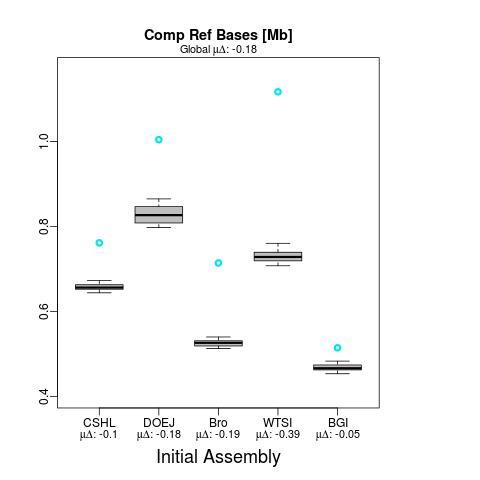


### Supplemental Figure 1f. Metassembly Compressed Reference Bases distribution.

Distributions of the number of Compressed Reference Bases (in mega bases) reported for the set of all 120 metassemblies, grouped by initial assembly. Blue circles indicate the corresponding value of the initial assembly. Below each initial assembly, the mean difference between the final metassembly and the initial assembly (μ𝚫) is shown in mega bases. The global mean difference is also shown at the top.


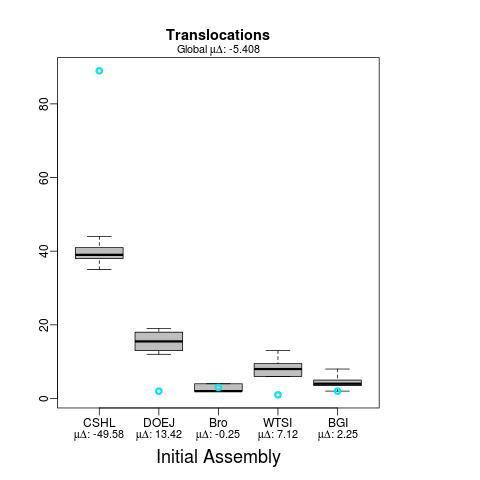


### Supplemental Figure 1g. Metassembly Translocations distribution.

Distributions of the number of Translocations reported for each of the 120 final metassemblies, grouped by initial assembly. Blue circles indicate the corresponding value of the initial assembly. Below each initial assembly, the mean difference between the final metassembly and the initial assembly (μ𝚫) is shown. The global mean difference is also shown at the top.


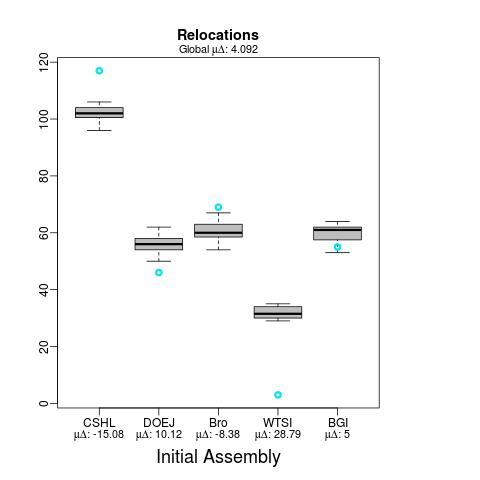


### Supplemental Figure 1h. Metassembly Relocations distribution.

Distributions of the number of Relocations reported for each of the 120 final metassemblies, grouped by initial assembly. Blue circles indicate the corresponding value of the initial assembly. Below each initial assembly, the mean difference between the final metassembly and the initial assembly (μ𝚫) is shown. The global mean difference is also shown at the top.


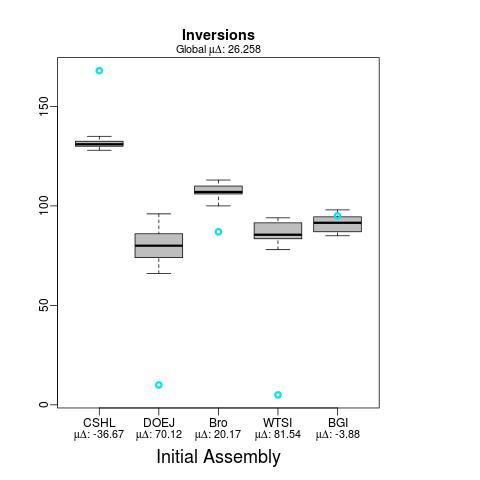


### Supplemental Figure 1i. Metassembly Inversions distribution.

Distributions of the number of Inversions reported for each of the 120 final metassemblies, grouped by initial assembly. Blue circles indicate the corresponding value of the initial assembly. Below each initial assembly, the mean difference between the final metassembly and the initial assembly (μ𝚫) is shown. The global mean difference is also shown at the top.


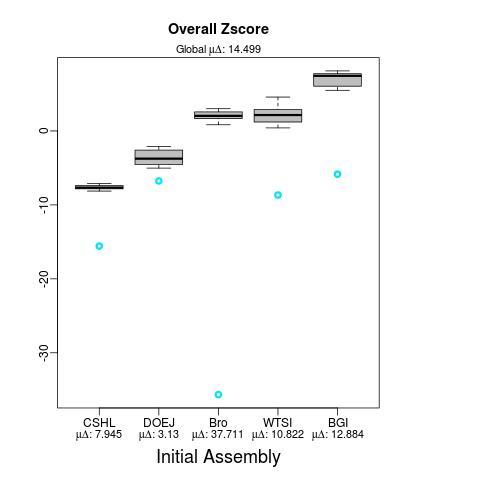


### Supplemental Figure 1j. Metassembly Overall Z-score distribution.

Distributions of Overall Z-scores computed for each of the 120 final metassemblies, grouped by initial assembly. Blue circles indicate the corresponding value of the initial assembly. Below each initial assembly, the mean difference between the final metassembly and the initial assembly (μ𝚫) is shown in mega bases. The global mean difference is also shown at the top.

## Supplemental Figure 2. Change in CE-statistic at indel breakpoint positions for the Assemblathon 1 metassemblies


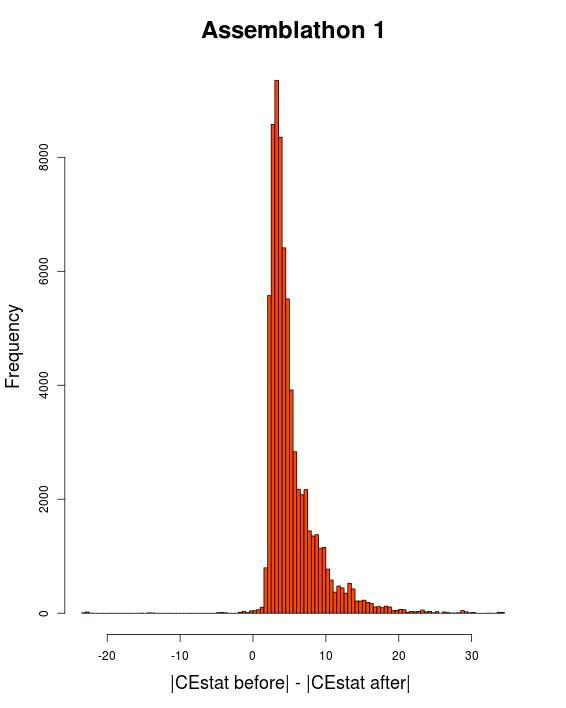


Histogram of the difference in CE-statistic before and after modifying the primary assembly. All insertion/deletion events processed in the 4 merging steps of all the 120 metassemblies are shown. Positive values indicate that the CE-statistic is improved upon the modification, suggesting that the changes made by our algorithm are correct.

## Supplemental Figure 3. Assemblathon 1 Comparison between Metassembler and GAM

##
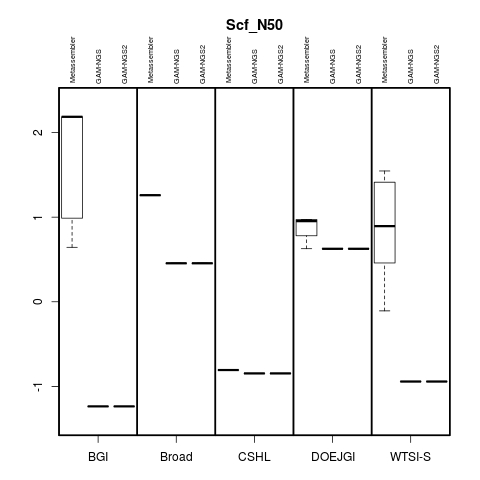


### Supplemental Figure 3a. Comparison of Metassembly and GAM-NGS scaffold NG50 size distribution.

Comparison of the distributions of scaffold NG50 Z-scores computed using all 360 final assemblies. For each algorithm the set of all the 120 metassemblies is shown grouped by initial assembly. Results for both sets of GAM-NGS input parameters are shown (GAM-NGS and GAM-NGS2).

##
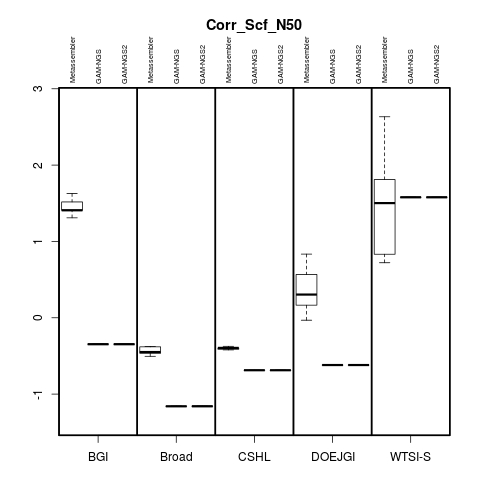


### Supplemental Figure 3b. Comparison of Metassembly and GAM-NGS GAGE-corrected scaffold NG50 Z-score distribution.

Comparison of the distributions of GAGE-corrected scaffold NG50 Z-scores computed using all 360 final assemblies. For each algorithm the set of all the 120 metassemblies is shown grouped by initial assembly. Results for both sets of GAM-NGS input parameters are shown (GAM-NGS and GAM-NGS2).

##
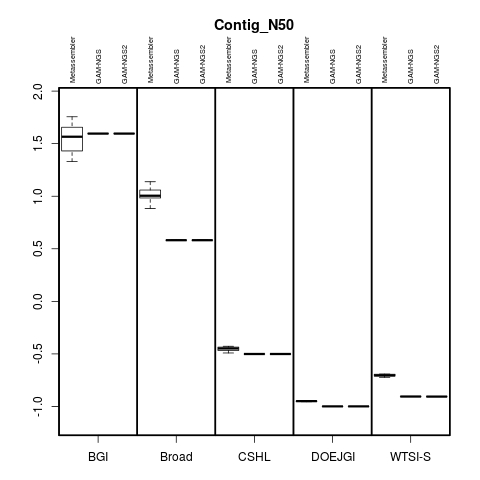


### Supplemental Figure 3c. Comparison of Metassembly and GAM-NGS contig NG50 Z-scores distribution.

Distributions of contig NG50 Z-scores computed using all 360 final assemblies. For each algorithm the set of all the 120 metassemblies is shown grouped by initial assembly. Results for both sets of GAM-NGS input parameters are shown (GAM-NGS and GAM-NGS2).


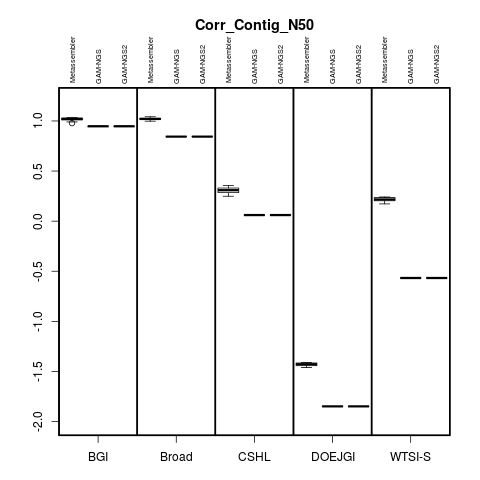


### Supplemental Figure 3d. Comparison of Metassembly and GAM-NGS GAGE-corrected contig NG50 Z-scores distribution.

Distributions of GAGE-corrected contig NG50 Z-scores computed using all 360 final assemblies. For each algorithm the set of all the 120 metassemblies is shown grouped by initial assembly. Results for both sets of GAM-NGS input parameters are shown (GAM-NGS and GAM-NGS2)


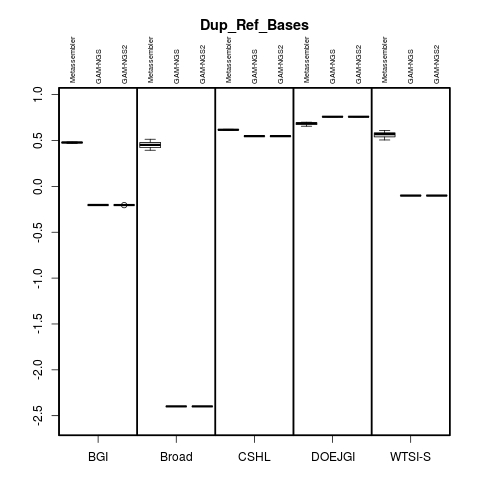


### Supplemental Figure 3e. Comparison of Metassembly and GAM-NGS Duplicated Reference Bases distribution.

Distributions of Z-scores for the number of Duplicated Reference Bases computed using all 360 final assemblies. For each algorithm the set of all the 120 metassemblies is shown grouped by initial assembly. Results for both sets of GAM-NGS input parameters are shown (GAM-NGS and GAM-NGS2)


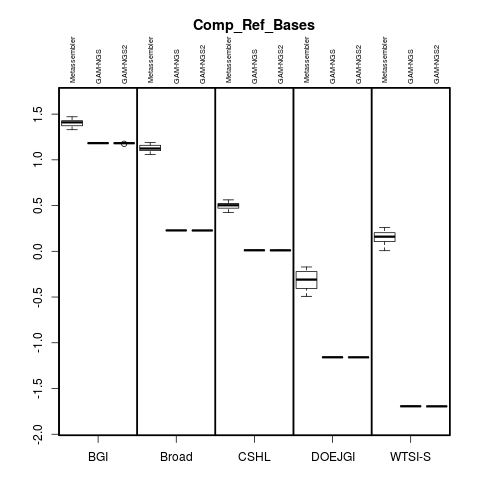


### Supplemental Figure 3f. Comparison of Metassembly and GAM-NGS Compressed Reference Bases distribution.

Distributions of Z-scores for the number of Compressed Reference Bases computed using all 360 final assemblies. For each algorithm the set of all the 120 metassemblies is shown grouped by initial assembly. Results for both sets of GAM-NGS input parameters are shown (GAM-NGS and GAM-NGS2).


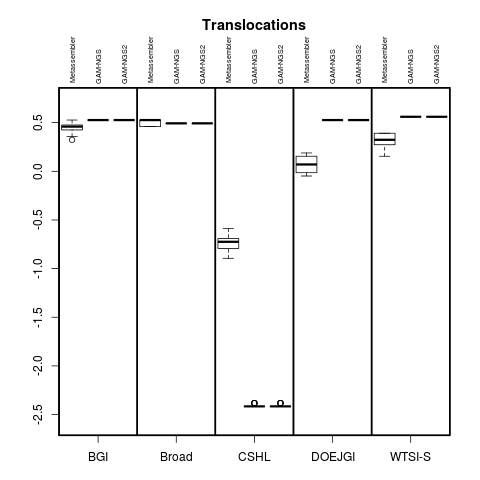


### Supplemental Figure 3g. Comparison of Metassembly and GAM-NGS Translocations distribution.

Distributions of Z-scores for the number of Translocations computed using all 360 final assemblies. For each algorithm the set of all the 120 metassemblies is shown grouped by initial assembly. Results for both sets of GAM-NGS input parameters are shown (GAM-NGS and GAM-NGS2).


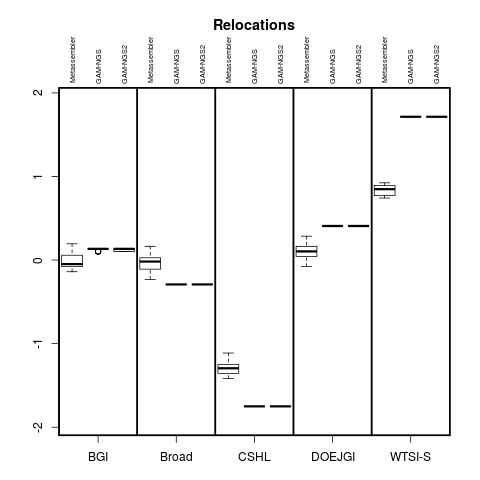


### Supplemental Figure 3h. Comparison of Metassembly and GAM-NGS Relocations distribution.

Distributions of Z-scores for the number of Relocations computed using all 360 final assemblies. For each algorithm the set of all the 120 metassemblies is shown grouped by initial assembly. Results for both sets of GAM-NGS input parameters are shown (GAM-NGS and GAM-NGS2).


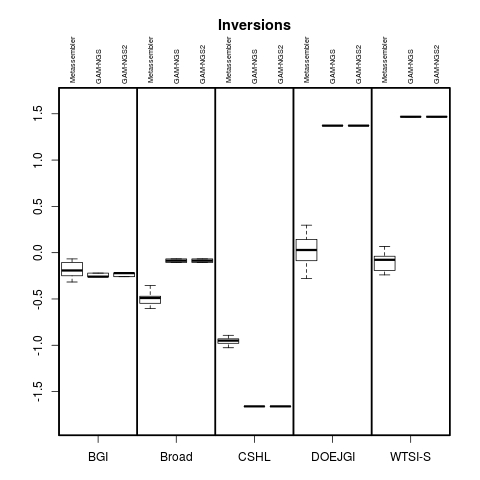


### Supplemental Figure 3i. Comparison of Metassembly and GAM-NGS Inversions distribution.

Distributions of Z-scores for the number of Inversions computed using all 360 final assemblies. For each algorithm the set of all the 120 metassemblies is shown grouped by initial assembly. Results for both sets of GAM-NGS input parameters are shown (GAM-NGS and GAM-NGS2).


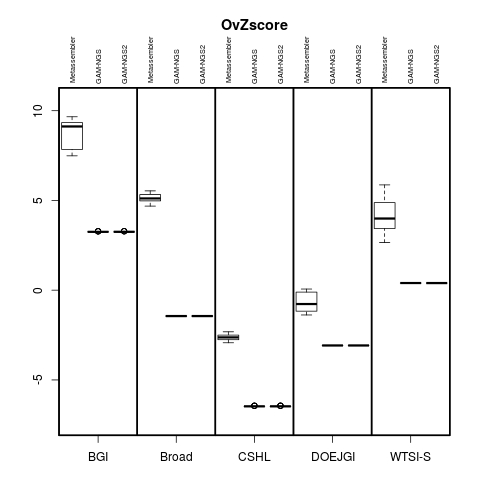


### Supplemental Figure 3j. Comparison of Metassembly and GAM-NGS Overall Z-score distribution.

Distributions of Overall Z-scores computed using all 360 final assemblies. For each algorithm the set of all the 120 metassemblies is shown grouped by initial assembly. Results for both sets of GAM-NGS input parameters are shown (GAM-NGS and GAM-NGS2).

##

## Supplemental Figure 4. Boxplots of Overall Z-scores computed using only ICA-selected metrics, for all Assemblathon 1 metassamblies.

##
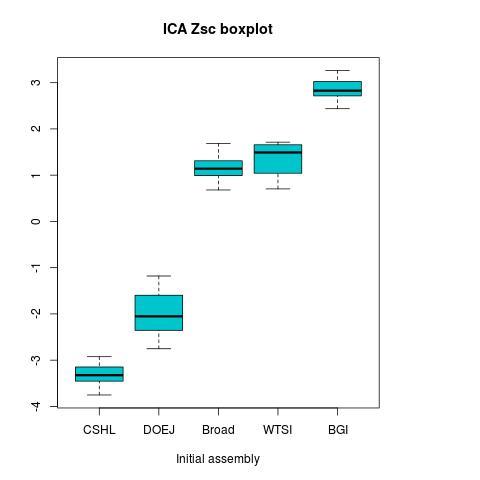


### Supplemental Figure 4a. Overall Z-scores using only the top 50% ICAs

Distributions of Overall Z-scores computed using only ICA-selected metrics from the top 50% of the ICAs in the kurtosis distribution. Boxplots for the set of all 120 metassemblies grouped by initial assembly are shown.


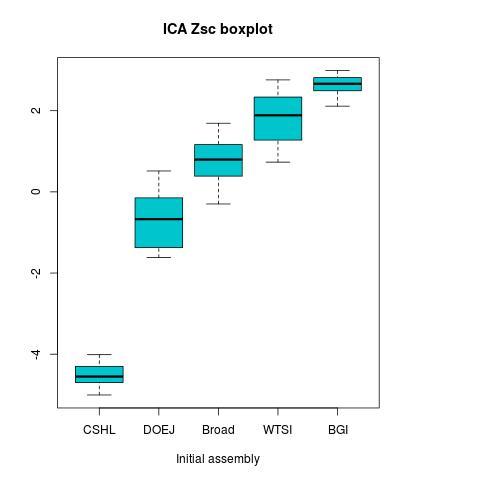


### Supplemental Figure 4b. Overall Z-scores using only the top 80% ICAs

Distributions of Overall Z-scores computed using only ICA-selected metrics from the top 80% of the ICAs in the kurtosis distribution. Boxplots for the set of all 120 metassemblies grouped by initial assembly are shown.

## Supplemental Figure 5. Assemblathon 2: Change in CE-statistic at indel breakpoint positions

### Supplemental Figure 5a. Fish: Change in CE-statistic at indel breakpoint positions


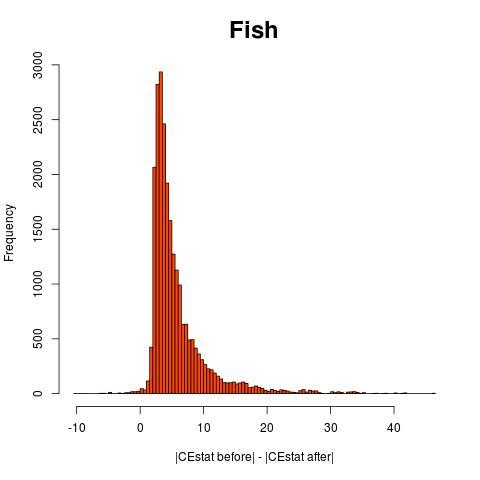


Histogram of the difference in CE-statistic before and after modifying the primary assembly. All insertion/deletion events processed in the 5 merging steps of the three metassemblies are shown. Positive values indicate that the CE-statistic is improved upon the modification, suggesting that the changes made by our algorithm are correct.

### Supplemental Figure 5b. Bird: Change in CE-statistic at indel breakpoint positions


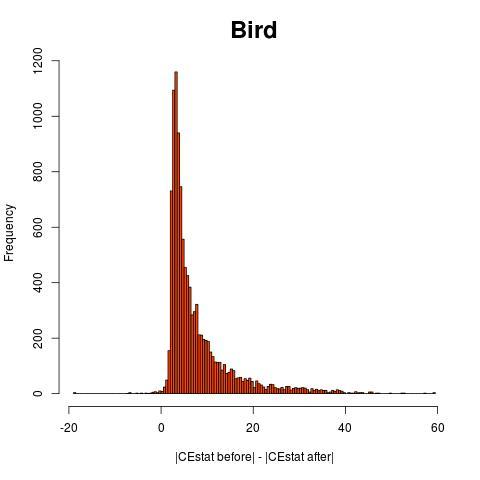


Histogram of the difference in CE-statistic before and after modifying the primary assembly. All insertion/deletion events processed in the 5 merging steps of the three metassemblies are shown. Positive values indicate that the CE-statistic is improved upon the modification, suggesting that the changes made by our algorithm are correct.

### Supplemental Figure 5c. Snake: Change in CE-statistic at indel breakpoint positions

###
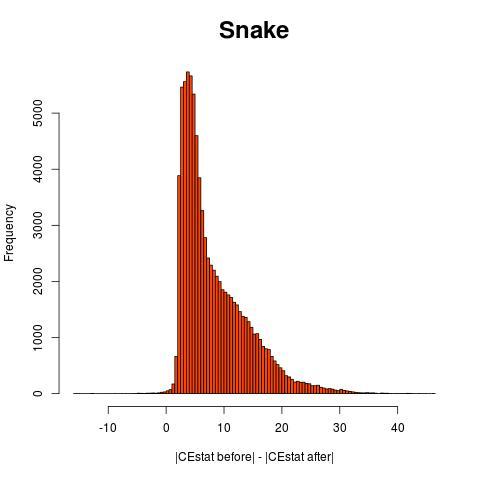


Histogram of the difference in CE-statistic before and after modifying the primary assembly. All insertion/deletion events processed in the 5 merging steps of the three metassemblies are shown. Positive values indicate that the CE-statistic is improved upon the modification, suggesting that the changes made by our algorithm are correct.
